# Ultrasonographic Evaluation of Solid Organ Sizes in Children with Primary Malnutrition: A Preliminary Study

**DOI:** 10.3390/jcm14010169

**Published:** 2024-12-31

**Authors:** Kamil Doğan, Şükrü Güngör, Adil Doğan, Seda Nida Karaküçük

**Affiliations:** 1 Radiology Department, Faculty of Medicine, Kahramanmaras Sutcu Imam University, 46050 Kahramanmaras, Türkiye; dradildogan@hotmail.com (A.D.); drsedanida@gmail.com (S.N.K.); 2 Pediatric Department, Faculty of Medicine, Kahramanmaras Sutcu Imam University, 46050 Kahramanmaras, Türkiye; sukru.gungor@yahoo.com

**Keywords:** malnutrition, liver, spleen, kidney, children

## Abstract

**Objectives:** Malnutrition is a common health problem affecting overall body functions, growth, and development. The aim of the present study was to explore any potential changes in solid organ sizes due to malnutrition and, if so, their correlation with the degree of malnutrition. **Materials and Methods:** Solid organ sizes (liver, spleen, and kidneys) in patients with primary malnutrition were measured prospectively using ultrasonography. **Results:** A correlation was observed between changes in liver, spleen, and kidney sizes and left kidney parenchymal thickness and the degree of malnutrition in patients. **Conclusions:** The presence and degree of malnutrition were directly proportional to significant decreases in organ sizes. The present study is the first to reveal a positive correlation between anthropometric measurement Z scores and organ sizes.

## 1. Introduction

Malnutrition is a systemic disease resulting from insufficient or unbalanced intake of micronutritional elements during growth and development, and it affects all organs negatively. Undernutrition continues to be the principal cause of ill health and premature mortality and morbidity among children in developing countries.

Primary malnutrition is common in developing countries with an insufficient food supply caused by various social, economic, and environmental factors [1,2].

Malnutrition reportedly causes many functional disorders in different systems, such as the heart, pancreas, immune system, thoracic muscle mass, subcutaneous fat, and thymus. It was also demonstrated that the expression of extracellular matrix proteins in rats is decreased with insufficient protein intake and causes the development of liver degeneration. Protein malnutrition induces structural changes, such as lymphoid organ atrophy, and increases spontaneous apoptotic cells in the spleen, which results in functional disorders of the immune system. Low glomerular filtration rates were reported in children with malnutrition. In addition, changes in the thymus size during malnutrition were analyzed via ultrasonography (USG) [3,4,5,6,7,8].

Abdominal USG is widely used to analyze different pediatric diseases and measure and monitor normal organ growth [9]. Various factors, such as age, height, weight, body surface area, and ethnicity, can affect an organ’s size [10]. Although functional studies on malnutrition in the liver, spleen, and kidneys have been performed, USG has not been employed to gain insight into structural changes in these organs.

The development of the above-mentioned functional systemic changes is likely to cause changes in organ sizes in pediatric patients with malnutrition. There is a lack of information on this subject in the literature; therefore, the aim of the present study was to explore any potential changes in solid organ sizes in pediatric patients with primary malnutrition using USG and, if so, their correlation with the degree of malnutrition.

## 2. Materials and Methods

Between April 2022 and June 2023, the organ sizes of patients diagnosed with primary malnutrition in the pediatric gastroenterology clinic of a tertiary university hospital were prospectively evaluated by a radiologist using USG. The data obtained were statistically compared with the gender- and age-matched healthy control group.

This study was approved by the Local Ethics Committee of the Faculty of Medicine of a local tertiary care university hospital (2022/14 decision no: 09, Decision: Positive). The study was conducted in accordance with the principles of the Declaration of Helsinki. Informed consent was obtained from the patients before the study.

**Exclusion Criteria**: Patients diagnosed with hepatosplenomegaly, cerebral palsy, well-known solid organ diseases (renal failure, chronic liver disease, depot disease, etc.), hematological malignancies, syndromic diseases (Down syndrome, Turner syndrome, etc.), anatomical defects, and underlying disease-causing secondary malnutrition (coeliac, cystic fibrosis, etc.) were not included in the present study.

**Control Group:** Patients with nonspecific abdominal pain who presented to the pediatric gastroenterology outpatient clinic and were age-, gender-, and ethnicity-compatible with the malnourished patient group and who had no chronic disease were prospectively and consecutively selected.

### 2.1. Evaluation of Malnutrition

*Height***:** Height was measured without shoes and socks using a calibrated vertical and portable stadiometer. Two-year-old or younger children were measured lying on a flat ground with an infantometer.

*Weight*: A digital electronic weight device set to the nearest decimal fraction of a kilogram was used to measure weight with light clothes.

Patients’ weight, height, and body mass index (BMI) Z-scores were calculated. Their age and gender were in accordance with the World Health Organization (WHO) data. Patients with a Z-score lower than −1 for any weight, height, or BMI parameters were diagnosed as suffering from malnutrition [11,12]. The degree of malnutrition was calculated as follows [13]: normal, weight-for-age Z-score > −1 SDS; mild underweight: weight-for-age Z-score ≥ −2 to ≤−1; moderate underweight, weight-for-age Z-score ≥ −3 to <−2; severe underweight, weight-for-age Z-score < −3.

### 2.2. Ultrasonographic Evaluation

All patients were subjected to USG using a GE Medical Systems Ultrasound LOGIQ E9 (GE LOGIQ E9 Ultrasound R5 XDClear, Chicago, IL, USA) with a 1–6 MHz convex probe, performed by a radiology specialist. Ultrasonography was performed by a radiologist with at least 15 years of experience.

**Figure 1:**
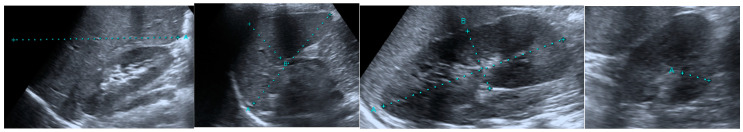
The technique used in this study for measuring solid organ dimensions sonographically is shown. Measurement of the craniocaudal dimension of the liver at the midclavicular line (**far left**), measurement of the long and short axis dimensions of the spleen (**second from the left**), measurement of the long dimension of the kidney (**second from the right**), and measurement of the kidney parenchymal thickness (**far right**).

*Liver*: For liver size measurements, USG is acknowledged as a user-dependent method [14], whereas computed tomography (CT) is the gold standard [15]. In the present study, a USG measurement method, for which performance was correlated with CT, was used [16,17], thus minimizing the user-dependent nature of the method. After the patient was placed in a supine position, a craniocaudal measurement was performed on the midclavicular line. In this plane, the screen image of the upper and inferior diaphragmatic end was taken without any superposition and gas artifacts. The mean value of three different measurements was considered (Figure 1).

*Spleen*: The patient was placed slightly in the right lateral decubitus position, and the probe was positioned on one of the lower intercostal spaces in the rear axial line. The longest image with the hilum was used for the measurement. The distance through the hilum and between the two ends was measured (Figure 1) [18].

*Kidney*: The probe was positioned supine on the patient’s lateral side. The patient was switched to a lateral decubitus position in the case of intestinal gas. The image was normalized with the patient holding their breath when it was difficult to evaluate the upper poles due to rib artifacts [19]. The middle part of the kidney was viewed in the short axis for the measurement of parenchymal thickness. Afterward, the mean value of three measurements on the deeper side was considered. The cortex and medulla were measured together (Figure 1).

### 2.3. Statistical Analysis

*Power analysis*: We found no studies on solid organ sizes in patients with malnutrition in the existing literature. Therefore, for an effect size of 0.5, a power of 0.95, and a critical t value of 1.65, the number of patients was at least 59 per group; the total number of patients was 118.

The Statistical Package for the Social Sciences for Windows 22 software was used for statistical analysis. Study variables were presented as the number (n) vs. percent (%) and mean ± standard deviation. The normal distribution of variables was tested using the Kolmogorov–Smirnov test. Normally distributed parameters were evaluated by performing a one-way analysis of variance or Student’s *t*-test, whereas Kruskal–Wallis or Mann–Whitney-U tests were used for numerical variables that did not show a normal distribution. Student’s *t*-tests, Mann–Whitney U tests, or chi-square tests were used to evaluate statistical significance. A one-way ANOVA test was used to determine whether there were statistically significant differences between the mean values of three or more independent groups. A correlation test was used to determine whether there was a linear relationship between two numerical measurements and, if so, the direction and severity of the relationship. The Pearson correlation coefficient was preferred if the data were normally distributed, whereas the Spearman Rank correlation coefficient was preferred if the data were not normally distributed. A *p*-value less than 0.05 was considered statistically significant.

## 3. Results

The mean age of all patients included in this study was 8.07 ± 5.54 (0.25–18) years. The mean age in the malnutrition group was 8.01 ± 5.92 (0.3–18) years, and in the healthy control group, it was 8.19 ± 4.81 (0.25–17.5) years. There was a predominance of 38 (58.5%) female patients in the malnutrition group and 72 (59%) in the control group. There was no statistically significant difference between the two groups in terms of age and gender (*p* = 0.977, *p* = 0.830, respectively).

**When the differences between the solid organ sizes** in the two groups were examined, right and left kidney length, right kidney thickness, and spleen and liver sizes were significantly lower in the malnutrition group than in the healthy control group (*p* < 0.001, *p* < 0.001, *p* = 0.016, *p* < 0.001, *p* < 0.001, respectively). Although left renal parenchymal thicknesses were lower in the malnutrition group, compared to those in the healthy control group, there was no statistically significant difference (*p* = 0.253) (Table 1).

**When the patients’ solid organ sizes were analyzed in terms of the degree of malnutrition** (Table 2), no statistically significant differences were observed among different age groups (*p* = 0.872). As expected, weight, height, and BMI Z-scores were lower in the malnutrition group (*p* < 0.001).

The patients’ right kidney, left kidney, liver, and spleen sizes were significantly lower **with different degrees of malnutrition** compared to those in the control group (*p* < 0.001, *p* = 0.002, *p* < 0.001, *p* < 0.001, respectively). However, no statistically significant differences were found between groups in terms of left kidney and right kidney parenchymal thicknesses (*p* = 0.251, *p* = 0.071, respectively).

**When the correlations between anthropometric measurement Z-scores and organ dimensions in the patients were analyzed** (Table 3), the right kidney size was moderately and positively correlated with weight and height Z-scores and weakly and positively correlated with the BMI Z-score (r = 0.366, *p* < 0.001; r = 0.416, *p* < 0.001; r = 0.200, *p* = 0.006, respectively). Similarly, the right kidney parenchymal thickness was weakly and positively correlated with weight, height, and BMI Z-scores (r = 0.226, *p* = 0.002; r = 0.217, *p* = 0.003; r = 0.157, *p* = 0.032, respectively). On the other hand, left kidney size was moderately and positively correlated with weight and height Z-scores but weakly and positively correlated with the BMI Z-score (r = 0.340, *p* < 0.001; r = 0.398, *p* < 0.001; r = 0.167, *p* = 0.023, respectively). The left kidney parenchymal thickness was very weakly and positively correlated with weight and height Z-scores but not with the BMI Z-score (r = 0.152, *p* = 0.039; r = 0.179, *p* = 0.015; r = 0.054, *p* = 0.469, respectively). The spleen size was moderately and positively correlated with weight and height Z-scores but weakly and positively correlated with the BMI Z-score (r = 0.442, *p* < 0.001; r = 0.425, *p* < 0.001; r = 0.297, *p* < 0.001, respectively). Finally, the liver size was moderately and positively correlated with weight and height Z-scores but weakly and positively correlated with the BMI Z-score (r = 0.339, *p* < 0.001; r = 0.345, *p* < 0.001; r = 0.173, *p* = 0.018, respectively).

**Figure 2:**
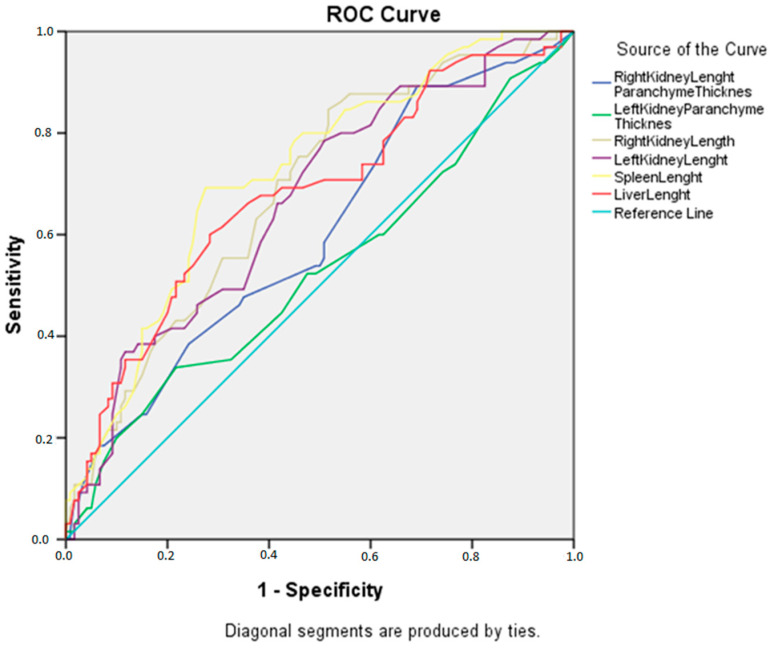
Analysis of results with ROC curve.

**When we investigated the cut-off values that could be used to indicate malnutrition in the solid organs measured in the study**, ROC curve analysis was performed to determine the best individual organ sizes to identify malnutrition (Figure 2). If the right kidney length was ≤65.5 mm, we showed that it could detect malnutrition with a 78.5% sensitivity and 48.8% specificity (*p* < 0.001). If the left kidney length was ≤72.5 mm, we showed that it could detect malnutrition with a 78.5% sensitivity and 49% specificity (*p* < 0.001). If the spleen length was ≤84.5 mm, we showed that it could detect malnutrition with a 69.2% sensitivity and 72.7% specificity (*p* < 0.001). If the liver length was ≤110.5 mm, we showed that it could detect malnutrition with a 60% sensitivity and 72% specificity (*p* < 0.001). If the right kidney parenchyma thickness was ≤7.75 mm, we showed that it could detect malnutrition with an 89.2% sensitivity and 31.4% specificity (*p* = 0.019). For the left kidney parenchyma thickness, we did not detect a significant cut-off point to detect malnutrition (*p* = 0.527) (Table 4).

**When we conducted a risk assessment for malnutrition using binary logistic regression analysis with these defined cut-off points**, we observed the following associations. A right kidney length of ≤65.5 was associated with a 5.323-fold increase in the risk of malnutrition, whereas a left kidney length of ≤72.5 mm was associated with a 3.643-fold increase in risk. Furthermore, a spleen length of ≤84.5 mm was associated with a 6.068-fold increase in the risk of malnutrition, and a liver length of ≤110.5 mm increased the risk by 3.882-fold. Additionally, a right kidney parenchyma thickness of ≤7.75 mm was associated with a 3.893-fold increase in the risk of malnutrition (Table 5).

**Based on a comprehensive evaluation of these risk factors, through multiple regression analysis**, we found that a reduced spleen size was associated with a significantly higher risk of malnutrition, when compared to other risk factors (*p* = 0.004), increasing the risk by 4.058-fold (95% confidence interval: 1.565–10.521).

## 4. Discussion

This study is the first to show that there is a significant decrease in solid organ dimensions in primary malnutrition. This demonstration was made with objective data based on Z scores. The liver, spleen, and kidneys, which are intra-abdominal structures that are particularly likely to be affected by malnutrition, were examined with multiple measurements in this study. The data obtained can be used in the diagnosis of malnutrition and can also be used safely in its follow-up. This discussion also explains in detail which additional studies will further enrich and strengthen the data obtained here.

There are multiple studies on functional liver, spleen, and kidney disorders in patients with malnutrition in the existing literature [7,20,21,22]. Unlike these, the present study focuses on structural changes, which is a less common research topic in the literature, aiming to reveal changes in solid organ sizes using USG in patients with malnutrition. Previous studies have reported decreased thymus sizes in patients with malnutrition [8], which coincides with the results of the present study, as all three solid organs displayed significant decreases in their sizes in the malnutrition group compared to those in the healthy control group.

It was demonstrated that malnutrition leads to a decrease in the extracellular matrix in the liver [20] and liver degeneration in rats [21]. These results could account for the decreased liver sizes observed in the malnutrition group in the present study.

It was also reported that malnutrition causes a decrease in the number of cells [23] and increased apoptosis [22] in the spleen. Both results correspond with those of the present study and are likely to be underlying reasons for the decreased spleen size observed in patients with malnutrition.

Kidney length, cortical thickness, and parenchymal thickness were found to be correlated with decreased renal function in patients with previous chronic kidney disease. As a result, kidney disease was considered the cause, while malnutrition was the effect [24]. In contrast, the present study indicated that the degree of malnutrition was correlated with a decrease in kidney size. However, the cause-and-effect relationship was not evident in the present study. It can still be suggested that there were changes in organ size secondary to malnutrition.

The functional features defined in the existing literature could form the basis of the mechanism related to the decrease in structural organ size. Further studies are needed to determine the possible role of previously reported data in the pathogenesis of a decreased structural organ size. Such a correlation has not been proven; however, it can be acknowledged as a probability. It is also of vital importance to analyze which functions are correlated with the structural changes observed in the present study.

Whether the degree of malnutrition is correlated with solid organ functional disorders has not been analyzed based on the existing literature. Although various results based on different degrees of malnutrition were reported in a few studies [8,25,26], they did not focus specifically on this correlation. On the other hand, the present study clearly demonstrated that solid organ sizes are positively correlated with weight, height, and BMI Z-scores, and that a more severe degree of malnutrition is directly proportional to a more apparent decrease in organ size. Despite such a positive correlation, transition values between different degrees of malnutrition were not defined in the present study, for which future studies can be conducted.

Sonographic measurements of organ sizes were evaluated with the help of the scales proposed in previous studies [27]. These scales can vary depending on different social regions [28,29]. Radiology specialists benefit from these scales to determine upper and lower organ size thresholds for different age groups. According to the results of the present study, decreased organ sizes in the malnutrition group, compared to those in the control group, definitely suggest a need to revise existing scales (if malnutrition is not an exclusion criterion) for the measurement of solid organ sizes.

Clinicians often employ anthropometric measurements for the diagnosis and monitoring of malnutrition [30]. Similarly, the present study found a correlation between anthropometric measurements and solid organ sizes, which offers a viable source of information for clinicians.

Whether decreased organ size is a cause or effect of malnutrition was not fully explored in the present study. A future study that analyzes children with a risk of malnutrition could find answers to this problem.

The main strength of the present study was that it included a relatively sufficient number of well-characterized patients with malnutrition and that an experienced radiology specialist performed USG scanning. However, considering the user-dependent nature of the USG [31], it will be challenging to compare the measurements from the present study to other researchers’ data. Since a single specialist was assigned to measure all data in the present study, the user-dependent nature of USG measurements did not pose a great problem.

Limitations: Being a user-dependent method, the USG measurement is a limitation of the present study. The integration of artificial intelligence-based systems into USG measurements will significantly contribute to user dependency. The fact that the present study focuses on a single user, a single clinic, and a certain number of patients is another limitation. Multi-centered future studies, which will be conducted by different users on more patients, are likely to offer more detailed data on the topic. In addition, since the present study also deals with changes in solid organ sizes, the restriction of measurements to two-dimensional images for three-dimensional structures, i.e., solid organs, also poses a limitation. In this respect, changes in volume will yield more accurate results.

The present study did not include data related to post-treatment monitoring. Future studies are recommended to focus on the post-treatment monitoring of the patients and any improvements in their organ sizes, including changes based on each degree of malnutrition. Thus, the obtained data can be used to assess treatment responses.

The present study focused on the correlation between kidney parenchymal thickness and malnutrition. Considering that the cortex and medulla are included within the parenchymal thickness, it is still unclear if or to what extent one of them is responsible for changes in organ sizes.

The patient group in the present study included those with primary malnutrition. Therefore, it cannot be argued that the obtained results can be generalized to different types of malnutrition, which again requires further studies on secondary malnutrition.

Although this study is a prospective study prepared in accordance with age and gender, it cannot be recommended to use the cut-off points related to organ sizes for all pediatric patients. In order to make a recommendation, each age group must be analyzed separately. For this reason, the number of patients in our study is insufficient.

## 5. Conclusions

The present study clearly indicated that the degree of malnutrition is directly proportional to a significant decrease in organ sizes, and it is the first study to show this. In addition, it is the first study to reveal a positive correlation between anthropometric measurement Z-scores and organ sizes. These data are likely to be useful for clinical practice and suggest that anthropometric measurement values should be taken into consideration for the evaluation of solid organ sizes. However, it should also be supported by further multi-centered studies based on a larger group of patients.

## Figures and Tables

**Table 1:** Evaluation of differences between the two groups in terms of organ sizes.

		Control (68)	Patient (122)	*p*
Right kidney	Length	85.58 ± 15.98	71.95 ± 19.58	<0.001
	Thickness	11.35 ± 3.33	9.93 ± 2.97	0.004
Left kidney	Length	89.24 ± 16.37	76.75 ± 19.30	<0.001
	Thickness	12.68 ± 5.23	11.57 ± 3.56	0.095
Spleen	Length	94.72 ± 20.53	75.58 ± 19.01	<0.001
Liver	Length	119.92 ± 26.29	99.84 ± 19.99	<0.001

Statistics: independent Student’s *t*-test.

**Table 2:** Evaluation of solid organ sizes according to the degree of malnutrition.

		Healthy	Malnutrition			*p*
			Mild	Moderate	Severe	
Age		9.35 ± 4.62	8.81 ± 5.31	8.98 ± 5.18	9.45 ± 5.67	0.941
Z-score	Weight	0.29 ± 0.92	−1.56 ± 0.28	−2.45 ± 0.28	−3.84 ± 0.85	<0.001
	Height	0.25 ± 0.88	−0.98 ± 1.04	−1.45 ± 1.10	−2.66 ± 1.23	<0.001
	BMI	0.14 ± 1.18	−1.37 ± 0.83	−2.15 ± 1.31	−2.96 ± 1.76	<0.001
Right kidney	Length	85.57 ± 15.98	74.09 ± 21.12	72.26 ± 18.07	68.13 ± 21.67	<0.001
	Thickness	11.34 ± 3.33	9.75 ± 2.95	10.11 ± 3.19	9.69 ± 2.39	0.033
Left kidney	Length	89.23 ± 16.36	78.53 ± 19.51	76.76 ± 18.44	74.14 ± 21.96	<0.001
	Thickness	12.68 ± 5.23	11.23 ± 3.28	11.90 ± 3.92	11.11 ± 2.82	0.303
Spleen	Length	94.73 ± 20.53	77.65 ± 19.35	76.90 ± 18.78	69.04 ± 18.60	<0.001
Liver	Length	119.92 ± 26.29	97.68 ± 21.32	101.34 ± 19.76	98.65 ± 19.26	<0.001

Statistics: one-way ANOVA; post-hoc tests, Scheffe test.

**Table 3:** Evaluation of the correlation between the patients’ anthropometric measurement Z-scores and organ sizes.

			Z-Score		
			Weight	Height	BMI
		N	190	190	190
Right kidney	Length	Pearson Correlation	0.387 **	0.466 **	0.219 **
		*p*	0.000	0.000	0.003
	Thickness	Pearson Correlation	0.270 **	0.274 **	0.205 **
		*p*	0.000	0.000	0.005
Left kidney	Length	Pearson Correlation	0.372 **	0.461 **	0.178 *
		*p*	0.000	0.000	0.016
	Thickness	Pearson Correlation	0.176 *	0.237 **	0.073
		*p*	0.018	0.001	0.326
Spleen	Length	Pearson Correlation	0.500 **	0.463 **	0.352 **
		*p*	0.000	0.000	0.000
Liver	Length	Pearson Correlation	0.426 **	0.441 **	0.232 **
		*p*	0.000	0.000	0.002

Statistics: correlation analysis, Pearson correlation. ** Correlation is significant at the 0.01 level; * Correlation is significant at the 0.05 level.

**Table 4:** Determining the best cut-off point of organ size to detect malnutrition.

	Cut-Off Point for Malnutrition	Sensitivity	Specificity	AUC	95% C.I.	*p*
Right kidney length *	≤65.5	0.846	0.488	0.687	0.609–0.765	<0.001
Left kidney length *	≤72.5	0.785	0.492	0.666	0.585–0.747	<0.001
Spleen length *	≤84.5	0.692	0.727	0.723	0.648–0.797	<0.001
Liver length *	≤110.5	0.600	0.719	0.681	0.599–0.762	<0.001
Right kidney parenchyme thicknes *	≤7.75	0.892	0.314	0.604	0.519–0.689	0.019

*: mm.

**Table 5:** Evaluation of risk factors for malnutrition by logistic regression analysis.

Risk Factors	OD	95% C.I.		*p*
		Lower	Upper	
Right kidney length (≤65.5 mm)	5.323	2.485	11.399	<0.001
Left kidney length (≤72.5 mm)	3.643	1.828	7.261	<0.001
Spleen lenght (≤84.5 mm	6.068	3.133	11.753	<0.001
Liver lenght (≤110.5 mm)	3.882	2.058	7.324	<0.001
Right kidney parenchyme thicknes (≤7.75 mm)	3.893	1.628	9.308	0.002
Statistics: Binary Logistic regression analysis				
Risk Factors	OD	95% C.I.		*p*
		Lower	Lower	
Right kidney length (≤65.5 mm)	3.237	0.910	0.910	0.070
Left kidney length (≤72.5 mm)	0.426	0.113	0.113	0.207
Spleen lenght (≤84.5 mm	4.058	1.565	1.565	0.004
Liver lenght (≤110.5 mm)	1.403	0.581	0.581	0.452
Right kidney parenchyme thicknes (≤7.75 mm)	1.332	0.466	0.466	0.592
Statistics: Multivariate Logistic Regression analysis				

## Data Availability

The original contributions presented in the study are included in the article, further inquiries can be directed to the corresponding authors.
